# Quantitative Microvascular Analysis With Wide-Field Optical Coherence Tomography Angiography in Eyes With Diabetic Retinopathy

**DOI:** 10.1001/jamanetworkopen.2019.19469

**Published:** 2020-01-17

**Authors:** Bingyao Tan, Jacqueline Chua, Emily Lin, Joyce Cheng, Alfred Gan, Xinwen Yao, Damon W. K. Wong, Charumathi Sabanayagam, Doric Wong, Choi Mun Chan, Tien Yin Wong, Leopold Schmetterer, Gavin S. Tan

**Affiliations:** 1 Singapore Eye Research Institute, Singapore National Eye Centre, Singapore; 2 Singapore Eye Research Institute–Nanyang Technological University Advanced Ocular Engineering (STANCE) Program, Singapore; 3 Academic Clinical Program, Duke–National University of Singapore Medical School, Singapore; 4 Department of Ophthalmology, Yong Loo Lin School of Medicine, National University of Singapore, Singapore; 5 Department of Ophthalmology, Lee Kong Chian School of Medicine, Nanyang Technological University, Singapore; 6 Department of Clinical Pharmacology, Medical University of Vienna, Vienna, Austria; 7 Center for Medical Physics and Biomedical Engineering, Medical University of Vienna, Vienna, Austria; 8 National University Health System, Singapore

## Abstract

**Importance:**

Wide-field optical coherence tomographic angiography (OCTA) may provide insights to peripheral capillary dropout in eyes with diabetic retinopathy (DR).

**Objective:**

To describe the diagnostic performance of wide-field OCTA with and without large vessel removal for assessment of DR in persons with diabetes.

**Design, Setting, and Participants:**

This case-control study was performed from April 26, 2018, to April 8, 2019, at a single tertiary eye center in Singapore. Case patients were those with type 2 diabetes for more than 5 years and bilateral DR diagnosed by fundus imaging; control participants included those with no self-reported history of diabetes, a fasting glucose level within the normal range in the past year, and no ocular pathologic findings. A wide-field (12 × 12-mm^2^) fovea-centered scan was performed using a prototype swept source OCTA system. Retinal microvasculature was examined by separating the angiograms into large vessels, capillaries, and capillary dropout regions.

**Main Outcomes and Measures:**

Area under the receiver operating characteristic curve (AUC) for DR severity discrimination using wide-field vascular metrics. Retinal perfusion density (RPD), capillary perfusion density (CPD), large vessel density (LVD), and capillary dropout density (CDD) were calculated. Low-contrast regions were excluded from the calculation.

**Results:**

A total of 49 eyes in 27 control participants (17 male [63.0%]; mean [SD] age, 59.96 [7.63] years; age range, 44-79 years) and 76 eyes in 47 patients with diabetes (29 male [61.7%]; mean [SD] age, 64.36 [8.08] years; range, 41-79 years) were included. Among eyes in patients with diabetes, 23 were in those with diabetes but no DR, 25 in those with mild nonproliferative DR, and 28 in those with moderate to severe nonproliferative DR. There was no difference in RPD, CPD, LVD, and CDD between the control group and the group with diabetes and no DR. There was a stepwise decrease in RPD, CPD, and CDD in the diabetes with no DR, mild nonproliferative DR, and moderate to severe nonproliferative DR groups, whereas LVD was not associated with DR staging. The nonproliferative DR group had decreased RPD, CPD, and CDD compared with the control group. The CPD had higher AUCs than RPD for discriminating diabetes with nonproliferative DR (combined mild and moderate to severe nonproliferative DR) vs no DM (AUC, 0.92 [95% CI, 0.87-0.98] vs 0.89 [95% CI, 0.83-0.95], *P* = .01), diabetes with no DR vs mild nonproliferative DR (AUC, 0.81 [95% CI, 0.68-0.94] vs 0.77 [95% CI, 0.64-0.91], *P* = .18), and mild nonproliferative DR vs moderate to severe nonproliferative DR (AUC, 0.82 [95% CI, 0.71-0.94] vs 0.78 [95% CI, 0.65-0.91], *P* = .01) but similar AUCs for no DM vs diabetes with no DR. The total perfusion density and CPD in wide-field OCTA had better discriminative power than the central 6 × 6-mm^2^ field (CPD, 0.89 [95% CI, 0.83-0.95] vs 0.84 [95% CI, 0.77-0.92], *P* = .06; total perfusion density, 0.93 [95% CI, 0.87-0.98] vs 0.90 [95% CI, 0.83-0.96], *P* = .06).

**Conclusions and Relevance:**

The findings suggest that wide-field OCTA provides information on microvascular perfusion and may be useful for detecting predominant peripheral capillary dropout in eyes with nonproliferative DR. A vascular selectivity approach excluding the large vessels may improve the discriminative power for different stages of DR.

## Key Points

**Question:**

What is the diagnostic performance of quantitative microvascular analysis using wide-field optical coherence tomographic angiography (OCTA) in eyes with diabetic retinopathy?

**Findings:**

In this case-control study of 49 eyes in 27 control participants and 76 eyes in 47 participants with diabetes, microvascular metrics obtained from wide-field OCTA accurately classified severity of nonproliferative diabetic retinopathy with high sensitivity.

**Meaning:**

The findings suggest that wide-field OCTA may be useful for assessment of microvascular status in eyes with diabetic retinopathy.

## Teaser

This case-control study analyzes the results obtained from wide-field optical coherence tomographic angiography (OCTA) with and without removal of large vessels to assess the presence and severity of diabetic retinopathy among individuals with diabetes.

## Introduction

Diabetic retinopathy (DR) is a common microvascular ocular complication of diabetes and is a leading cause of blindness in the working-age population.^1,2^ The population with diabetes globally is estimated to reach 366 million in 2030, with 34.6% having DR and 7% having vision-threatening DR.^3,4^

Optical coherence tomography angiography (OCTA) has advanced the understanding of DR and is used as a tool for detecting capillary nonperfusion.^5^ Traditionally, nonperfusion areas could only be detected by fluorescein angiography, but OCTA is a noninvasive, rapid, and simple approach to provide a 3-dimensional representation of the retinal vascular network.^6,7,8^ Vascular metrics, such as properties of fovea avascular zone, perfusion density, vessel density, and vascular fractal dimension have been reported in the context of diabetes or DR.^9,10,11,12,13,14,15,16^

Most current OCTA machines can image only a small field of view around the macula and provide little insight to the peripheral regions. Because vascular alterations occur predominantly in the peripheral area of the eye in DR,^17,18,19^ solely looking into a small field of view may result in misclassification (ie, eyes with poor peripheral perfusion may not be detected). Large field of view protocols have been recently adopted in several machines, and by stitching or adding an ocular lens, one can achieve a larger field of view up to a 20 × 20-mm^2^ field^20,21,22^; however, these methods have yet to be validated in larger populations.

**Figure 1.**
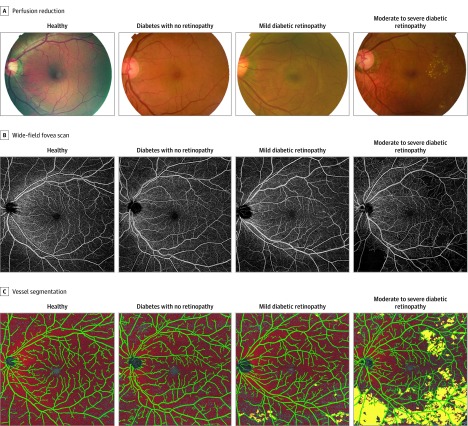
Representative Images of Eyes With Different Diabetic Retinopathy Severities

In this study, we investigated retinal microvascular changes using a wide-field OCTA scan (12 × 12-mm^2^ field) in patients with diabetes with or without DR compared with individuals without diabetes or ocular diseases. The wide-field images were further divided into a central 6 × 6-mm^2^ field and the remaining square annulus regions (Figure 1) to compare the wide-field protocol with traditional small field of view protocols. Localized capillary metrics were studied by uniformly dividing the entire field into 5 × 5 nonoverlapping blocks. We further hypothesized that large vessel removal from OCTA images would be associated with improved diagnostic performance of OCTA.

## Methods

### Study Participants

This cross-sectional case-control study was approved by the SingHealth Centralized institutional review board, Singapore, and conducted in accordance with the Declaration of Helsinki.^23^ Written informed consent was obtained from all participants. This study followed the Strengthening the Reporting of Observational Studies in Epidemiology (STROBE) reporting guideline. Patients with diabetes and DR were compared with patients with diabetes without DR and patients without diabetes (controls). The study was performed from April 26, 2018, to April 8, 2019, at a single tertiary eye center, the Singapore National Eye Center (SNEC), Singapore. Participants meeting inclusion criteria were patients who had type 2 diabetes for more than 5 years and bilateral nonproliferative DR (NPDR) that was diagnosed by fundus imaging according to the Early Treatment Diabetic Retinopathy Study DR grading scale by the SNEC Ocular Reading Centre.^24^ Diabetes criteria were a known physician diagnosis of type 2 diabetes and receipt of oral hypoglycemic agents or insulin therapy. Exclusion criteria were glaucoma, age-related macular degeneration, ocular opacity, or history of panretinal photocoagulation. Inclusion criteria for the control participants were no self-reported history of diabetes, a fasting glucose level within the normal range in the previous year, and no ocular pathologic findings (including glaucoma, age-related macular degeneration, or ocular opacity).

Another small group of participants (n = 12) was included from a study under the Prevention of and Intervention for Eye Diseases in the Elderly (PROVIDE) program.^25^ The results obtained for those participants were used to test the reproducibility of the measurements.

### Optical Coherence Tomography Angiography

Optical coherence tomography angiography is a noninvasive imaging modality that provides 3-dimensional structural and angiographic information of the posterior pole of the retina. The prototype swept source OCT (SS-OCT) system (PlexElite 9000; Zeiss Meditec) uses a wavelength scanning laser (central wavelength [λ_c_] = 1050 nm) as light source, and the spectral information is acquired by a photodetector. The system operation speed is dependent on the scanning rate of the swept source (100 000 amplitude scan [A-scan] per second), and the axial and lateral resolutions in tissue are 6.3 μm and 20 μm, respectively.

All the participants were scanned by the same trained ophthalmic technician (E.L.). A wide-field area (12 × 12-mm^2^ field) centered at the fovea was scanned, and each data volume consisted of 500 A-scans and 500 B-scans. Each B-scan was repeated twice to generate OCTA images using an optical microangiography algorithm.^26^ Motion-related artifacts were minimized by an integrated line-scanning ophthalmoscope eye tracker during data acquisition. A review software, PlexElite Review Software, version 1.6 (Zeiss Meditec), provided automated segmentation of retinal layers, and the inner retina was defined from inner limiting membrane to 70 μm above the retinal pigment epithelium. En face maximum projection angiographic images including superficial and deep vascular plexuses were extracted for further analysis. In the present study, superficial and deep vascular plexuses were not separated because of insufficient performance of the projection artifact removal algorithm in the wide-field images. For the reproducibility analysis, 1 eye was randomly chosen from the 12 PROVIDE participants and scanned twice by the same ophthalmic technician with an interval of 5 minutes between scans.

### Image Analysis

A custom MatLab algorithm (The MathWorks Inc) was developed for quantitative analysis. First, a combined Gabor and Hessian–based vessel filter was applied to enhance the contrast of large blood vessels, followed by a thresholding method to generate a binarization mask of the large vessels. Second, a sliding window scheme (window size, 3 × 3 mm^2^; sliding step, 1.5 mm) was used for segmenting capillaries. In each window, the threshold was empirically set to be 0.35 of the mean intensity of the large vessels. Third, all the segmented large vessel and capillary regions were set to 1 and the background was set to 0; the image contrast was then flipped by an inverse transform, and nonperfusion area was considered as 1. Intercapillary regions more than 0.36 mm^2^ were considered as capillary dropout regions. The foveal avascular zone and optic disc area were excluded manually from the capillary dropout calculation. The low-signal-related artifacts were filtered out based on the corresponding structural images.^27^ The following 4 vascular metrics were computed: (1) retinal total perfusion density (TPD; total perfused area per total imaged area), capillary perfusion density (CPD; capillary perfused area per total imaged area), large vessel density (LVD; large vessel area per total imaged area), and capillary dropout density (CDD; capillary dropout area per total imaged area). For TPD, CPD, and LVD, lower values suggest worse vascular disruption. For CDD, higher values suggest worse vascular disruption.

To study localized vascular changes, the wide-field 12 × 12-mm^2^ images were separated into (1) a central 6 × 6-mm^2^ field and (2) the remaining square annulus consisting of 5 × 5 nonoverlapping uniform blocks with a size of 2.4 × 2.4 mm^2^. The 4 vascular metrics (TPD, CPD, LVD, and CDD) were calculated in each subregion.

### Statistical Analysis

Data were analyzed using Stata, version 15.0 (StataCorp LLC) and R, version 3.5.0 (R Project for Statistical Computing). The repeatability of the measurement of each vascular metric was assessed using the intraclass correlation coefficient (ICC). The ICC values were defined as follows: less than 0.50 (poor repeatability), between 0.50 and 0.75 (moderate repeatability), between 0.75 and 0.90 (good repeatability), and greater than 0.90 (excellent repeatability).^28^

We compared 4 vascular metrics between the 3 DR severity groups (no DR, mild NPDR, and moderate to severe NPDR). The sampling distribution of the mean pairwise difference was obtained by nonparametric bootstrapping (1000 replicates) of individuals as the resampling clusters. This approach was taken to account for skewness of the distribution of certain vascular parameters and between-eye correlation of measurements made in an individual. The false discovery rate was controlled at a level of 5% using the Benjamini-Hochberg procedure to adjust *P* values (2-sided *P* < .05 was considered statistically significant) for multiple pairwise comparisons across vascular parameters and combinations of DR severity groups. We also conducted a post hoc test of the linear orthogonal contrast, the result of which is referred to as *P* value for trend to access the monotonical increase or decrease of the vascular metrics in different retinopathy status. We assessed the performance of TPD and CPD in discriminating control eyes and eyes with NPDR using the receiver-operating characteristic (ROC) curve. Comparisons of areas under the ROC curve (AUCs) were performed using an algorithm proposed by DeLong et al.^29^

## Results

### Patient Characteristics

A summary of patient characteristics is shown in Table 1. The initial quality check of wide-field OCTA excluded 30% to 40% of these scans because of multiple artifacts, such as low contrast, extensive motion artifacts, layer segmentation errors, and fixation problems. In the control group, a total of 49 eyes from 27 participants were included (17 male [63.0%]; mean [SD] age, 59.96 [7.63] years; age range, 44-79 years). In the group with diabetes, a total of 76 eyes from 47 participants were included (29 male [61.7%]; mean [SD] age, 64.36 [8.08] years; age range, 41-79 years). The DR severity was classified into 3 classes: no DR (23 eyes [30.2%]), mild NPDR (25 eyes [32.9%]), and moderate to severe NPDR (28 eyes [36.8%]). Higher glycated hemoglobin level was associated with DR severity. There was no difference in age, sex, serum glucose level, serum creatinine level, duration of diabetes, cholesterol level, high-density lipoprotein cholesterol level, triglyceride levels, low-density lipoprotein cholesterol level, and cholesterol ratio among groups, but systolic blood pressure was higher in the diabetes group.

**Table 1.** Characteristics of Study Participants by Diabetes and DR Status

Characteristic	Control Participants (n = 27)	Participants With Diabetes			*P* Value^a^
		No DR (n = 14)	Mild NPDR (n = 13)	Moderate to Severe NPDR (n = 20)	
Eyes, No. (%)	49 (100)	23 (30.2)	25 (32.9)	28 (36.8)	NA
Male, No. (%)	17 (63.0)	9 (64.3)	8 (61.5)	12 (60.0)	.99
Age, mean (SD), y	59.96 (7.63)	67.07 (6.93)	62.54 (6.38)	63.65 (8.22)	.06
Diabetes, No. (%)	0	14 (100)	13 (100)	20 (100)	<.001
Hypertension, No. (%)	10 (37.0)	13 (92.8)	10 (71.4)	15 (75.0)	<.001
Diabetes duration, mean (SD), y	0	16.50 (9.58)	26.23 (23.94)	19.90 (9.36)	.24
Creatinine, mean (SD), mg/dL	NA	0.94 (0.24)	0.98 (0.31)	1.05 (0.65)	.78
Glucose, mean (SD), mg/dL	NA	127.03 (66.85)	162.52 (82.89)	205.23 (90.27)	.07
Total cholesterol, mean (SD), mg/dL	NA	162.93 (37.84)	145.17 (31.27)	169.11 (33.98)	.15
High-density lipoprotein cholesterol, mean (SD), mg/dL	NA	51.73 (10.04)	47.88 (121.74)	47.88 (8.88)	.56
Low-density lipoprotein cholesterol, mean (SD), mg/dL	NA	94.59 (33.59)	76.06 (18.53)	95.75 (26.64)	.10
Triglycerides, mean (SD), mg/dL	NA	142.48 (53.10)	164.60 (97.35)	194.69 (100.89)	.26
Cholesterol ratio	NA	3.21 (0.75)	3.12 (0.60)	3.60 (0.79)	.14
Blood pressure, mm Hg					
Systolic	126.23 (17.60)	149.75 (25.30)	136.47 (17.58)	147.60 (21.46)	<.001
Diastolic	76.94 (6.67)	74.88 (11.71)	71.49 (11.08)	68.53 (10.60)	.02
Glycated hemoglobin, %	NA	6.76 (1.17)	7.42 (0.81)	8.83 (2.00)	<.001

Abbreviations: DR, diabetic retinopathy; NA, not applicable; NPDR, nonproliferative diabetic retinopathy.

SI conversion: To convert total, high-density lipoprotein, and low-density lipoprotein cholesterol levels to millimoles per liter, multiply by 0.0259; plasma creatinine to micromoles per liter, multiply by 88.4; glucose to millimoles per liter, multiply by 0.0555; to convert triglycerides to millimoles per liter, multiply by 0.0113; and glycated hemoglobin to proportion of total hemoglobin, multiply by 0.01.

^a^ Analysis of variance was used to compare continuous variables, and χ^2^ test was used to compare categorical variables.

### Repeatability

The repeatability test was done for 12 patients with a mean (SD) age of 70.12 (6.68) years (range, 60-84 years). Six of the participants had diabetes, and 5 had systemic hypertension. The ICC scores were 0.80 for TPD, 0.81 for CPD, 0.80 for LVD, and 0.92 for CCD. In the cropped, central 6 × 6-mm^2^ field, ICC scores were lower (0.41 for TPD, 0.71 for CPD, and 0.49 for LVD). In the square annulus, area reproducibility was better, and the ICC scores were 0.84 for TPD, 0.81 for CPD, 0.90 for LVD, and 0.93 for CCD.

Examples of fundus images, OCTA images, and processed images of patients in the control, no DR, mild NPDR, and moderate to severe NPDR groups are shown in Figure 1. The OCTA images showed decreased capillary perfusion and increased capillary dropout area associated with worsening DR severity, and the capillary dropout regions were located mainly in the peripheral parts of the angiogram.

### Quantitative Analysis

The quantitative analysis is summarized in Table 2, and the *P* values from statistical tests are summarized in Table 3. In a comparison between any DR and no diabetes using wide-field scans, mean (SD) TPD and CPD decreased (TPD: 68.48% [11.15%] vs 85.91% [3.84%], *P* < .001; CPD: 51.51% [11.37%] vs 70.67% [3.67%], *P* < .001), whereas mean (SD) LVD and CDD increased (LVD: 16.94% [1.75%] vs 15.20% [1.00%], *P* < .001; CDD: 11.21% [8.39%] vs 0.42% [0.51%], *P* < .001). There was no difference in the OCTA metrics between the no DR and no diabetes groups. In a comparison of the mild NPDR group with the no DR groups, TPD and CPD decreased (TPD: –0.85 [95% CI, –1.33 to –0.30], *P* = .003; CPD: –0.89 [95% CI, –1.38 to –0.32], *P* = .002), whereas CDD increased (0.51 [95% CI, 0.07-0.91], *P* = .03). For the moderate to severe NPDR group vs the mild NPDR group, TPD and CPD decreased (TPD: –0.96 [95% CI, –1.49 to –0.45], *P* = .001; CPD: –1.01, [95 % CI, –1.46 to –0.59], *P* < .001), whereas CDD increased (1.07 [95% CI, 0.46-1.77], *P* = .002); there was no difference in LVD (0.67 [95% CI, 0.02-1.29], *P* = .06).

**Table 2.** Result of Optical Coherence Tomographic Angiography Analysis in Participants by Diabetes and DR Status

Parameter	Value, Mean (SD)				
	Control Participants	Participants With Diabetes and No DR	Participants With Nonproliferative DR		
			All	Mild	Moderate to Severe
**Wide Field (12 × 12 mm^2^)**					
Total perfusion density	85.91 (3.84)	86.71 (7.52)	68.48 (11.15)	76.18 (9.48)	61.62 (7.42)
Capillary perfusion density	70.67 (3.67)	71.33 (8.42)	51.51 (11.37)	59.90 (8.82)	44.01 (7.48)
Large vessel density	15.20 (1.00)	15.34 (1.51)	16.94 (1.75)	16.24 (1.55)	17.56 (1.69)
Capillary dropout density	0.42 (0.51)	1.51 (2.59)	11.21 (8.39)	5.65 (6.13)	16.17 (6.89)
**Central (6 × 6 mm^2^)**					
Total perfusion density	93.01 (3.33)	92.67 (5.14)	80.59 (9.12)	86.30 (7.70)	75.49 (7.03)
Capillary perfusion density	78.39 (3.51)	77.88 (5.89)	63.86 (9.48)	70.69 (6.97)	57.76 (6.93)
Large vessel density	14.62 (1.44)	14.80 (1.49)	16.73 (2.11)	15.61 (1.56)	17.73 (2.03)
Capillary dropout density	0.06 (0.22)	0.16 (0.37)	2.12 (2.62)	0.91 (1.58)	3.21 (2.87)
**Square Annulus**					
Total perfusion density	83.52 (4.34)	84.70 (8.39)	64.39 (12.22)	72.73 (10.27)	56.94 (8.41)
Capillary perfusion density	68.07 (4.09)	69.12 (9.31)	47.32 (12.30)	56.23 (9.57)	39.37 (8.41)
Large vessel density	15.39 (0.96)	15.52 (1.66)	17.01 (1.78)	16.45 (1.72)	17.51 (1.67)
Capillary dropout density	0.54 (0.67)	1.96 (3.37)	14.24 (10.66)	7.23 (7.78)	20.50 (8.84)

Abbreviation: DR, diabetic retinopathy.

**Table 3.** Comparison of Vascular Density Parameters Between DR Severity Groups^a^

Parameter	Any DR vs Control		No DR vs Control		Mild NPDR vs No DR		Moderate to Severe NPDR vs Mild NPDR		*P* Value for Trend
	Difference (95% CI)^b^	*P* Value^c^	Difference (95% CI)^b^	*P* Value^c^	Difference (95% CI)^b^	*P* Value^c^	Difference (95% CI)^b^	*P* Value^c^	
**Wide Field (12 × 12 mm^2^)**									
Total perfusion density	−1.27 (−1.62 to −0.91)	<.001	0.09 (−0.41 to 0.48)	.79	−0.85 (−1.33 to −0.30)	.003	−0.96 (−1.49 to −0.45)	.001	<.001
Capillary perfusion density	−1.36 (−1.66 to −1.02)	<.001	0.07 (−0.48 to 0.47)	.81	−0.89 (−1.38 to −0.32)	.002	−1.01 (−1.46 to −0.59)	<.001	<.001
Large vessel density	1.00 (0.58 to 1.38)	<.001	0.08 (−0.36 to 0.66)	.81	0.56 (−0.08 to 1.14)	.10	0.67 (0.02 to 1.29)	.06	<.001
Capillary dropout density	1.29 (0.92 to 1.70)	<.001	0.21 (0 to 0.60)	.18	0.51 (0.07 to 0.91)	.03	1.07 (0.46 to 1.77)	.002	<.001
**Central (6 × 6 mm^2^)**									
Total perfusion density	−1.14 (−1.56 to −0.79)	<.001	−0.05 (−0.52 to 0.31)	.81	−0.61 (−1.09 to −0.12)	.02	−0.90 (−1.50 to −0.28)	.007	<.001
Capillary perfusion density	−1.28 (−1.64 to −0.95)	<.001	−0.07 (−0.57 to 0.31)	.81	−0.66 (−1.11 to −0.14)	.02	−1.05 (−1.56 to −0.56)	<.001	<.001
Large vessel density	0.98 (0.54 to 1.40)	<.001	0.09 (−0.34 to 0.55)	.79	0.40 (−0.10 to 0.93)	.18	0.92 (0.35 to 1.52)	.004	<.001
Capillary dropout density	0.92 (0.56 to 1.30)	<.001	0.09 (−0.05 to 0.34)	.41	0.39 (0.03 to 0.97)	.13	0.83 (0.15 to 1.52)	.03	<.001
**Square Annulus**									
Total perfusion density	−1.27 (−1.62 to −0.92)	<.001	0.11 (−0.38 to 0.50)	.74	−0.89 (−1.37 to −0.35)	.002	−0.94 (−1.46 to −0.43)	.001	<.001
Capillary perfusion density	−1.35 (−1.66 to −1.03)	<.001	0.10 (−0.46 to 0.50)	.79	−0.93 (−1.42 to −0.36)	.002	−0.98 (−1.45 to −0.55)	<.001	<.001
Large vessel density	0.93 (0.54 to 1.31)	<.001	0.08 (−0.41 to 0.70)	.81	0.58 (−0.08 to 1.21)	.12	0.52 (−0.18 to 1.16)	.18	<.001
Capillary dropout density	1.29 (0.92 to 1.70)	<.001	0.22 (0 to 0.61)	.18	0.51 (0.06 to 0.91)	.03	1.06 (0.45 to 1.76)	.002	<.001

Abbreviations: DR, diabetic retinopathy; NPDR, nonproliferative diabetic retinopathy.

^a^ Each parameter was normalized by subtracting the mean and dividing by the SD for comparability across parameters.

^b^ Nonparametric bootstrapping (1000 replicates) with individuals as the resampling clusters (75 individuals, 125 eyes) was used to obtain 95% CIs.

^c^ Benjamini-Hochberg–corrected *P* value for multiple testing.

The square annulus and the central 6 × 6-mm^2^ field showed similar differences between the any DR and no diabetes groups, but the square annulus showed greater OCTA differences than the central 6 × 6-mm^2^ field. Specifically, when comparing mild NPDR and no DR groups, CDD was not different in the central 6 × 6-mm^2^ field but was significantly increased in the square annulus (mean difference in units of SD, 0.51 [95% CI, 0.06-0.91]; *P* = .03), indicating that capillary dropout occurred predominantly in the peripheral regions during the early stages of NPDR.

### Qualitative Localized Dropout

**Figure 2.**
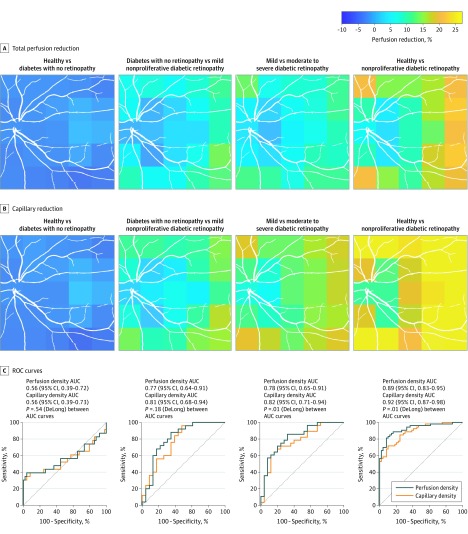
Perfusion Density Change and Discriminative Power in Stratified Diabetic Retinopathy Severities Receiver operating characteristic (ROC) curves for discriminating diabetic retinopathy severities using total perfusion density and capillary perfusion density. AUC indicates area under the ROC curve.

Figure 2A and B demonstrate localized capillary dropout associated with DR severity. The color in each of the 5 × 5-mm^2^ blocks represents the relative perfusion reduction. The capillary difference between the no DR and no diabetes groups was minimal. Mild NPDR was associated with subtle capillary dropout, and the change was uniformly distributed over the entire 12 × 12-mm^2^ field. The capillary dropout in the moderate to severe NPDR group mainly occurred in the periphery and most often in the temporal quadrant.

Figure 2C shows the ROC curves for TPD and CPD for separation between DR severities and between controls and persons with NPDR. In a comparison between controls and those without DR, there was no difference between TPD and CPD in AUCs. By contrast, the AUCs of CPD were larger than those of TPD when comparing diabetes with no DR vs mild NPDR (AUC, 0.81 [95% CI, 0.68-0.94] vs 0.77 [95% CI, 0.64-0.91], *P* = .18), or mild NPDR vs moderate to severe NPDR (AUC, 0.82 [95% CI, 0.71-0.94] vs 0.78 [95% CI, 0.65-0.91], *P* = .01). Furthermore, CPD had a higher AUC than TPD when comparing eyes with NPDR (combined mild and moderate to severe NPDR) and control eyes (AUC, 0.92 [95% CI, 0.87-0.98] vs 0.89 [95% CI, 0.83-0.95], *P* = .01).

The AUCs of TPD and CPP in the regions of 6 × 6-mm^2^ field and the remaining square annulus were also calculated. The TPD and CPP in wide-field OCTA had better discriminative power than the central 6 × 6-mm^2^ field (CPP: 0.89 [95% CI, 0.83-0.95] vs 0.84 [95% CI, 0.77-0.92], *P* = .06; TPD: 0.93 [95% CI, 0.87-0.98] vs 0.90 [95% CI, 0.83-0.96], *P* = .06), although this difference was not statistically significant. The AUCs were similar between the wide field (12 × 12-mm^2^ field) and the square annulus.

## Discussion

In this article, we describe results of several quantitative microvascular metrics using a protocol of wide-field OCTA in patients with DR. The key findings of the present study are that wide-field OCTA may be useful to detect predominant peripheral capillary dropout in eyes with NPDR.

Furthermore, we showed that separating the angiograms into large vessels, capillaries, and capillary dropout regions may improve the diagnostic performance of OCTA in discriminating the different stages of DR. Because many of the alterations in the moderate to severe stage of NPDR were found to be peripheral in the present study, wide-field OCT appears to be better suitable than a 3 × 3-mm^2^ or 6 × 6-mm^2^ field OCTA to detect areas of capillary loss.

Our results were in agreement with several previous reports^20,21^ involving wide-field imaging modalities, including fluorescein angiography and OCTA. By stitching several fields together^21^ or implementing an extra lens to expand the scanning area,^20^ prevalent peripheral capillary dropout in DR was reported. Imaging far periphery vascularization is technically difficult. Using an ultrafast Multi-Megahertz OCT system (Optores GmbH) and special ultrawide-field ophthalmic optics design can potentially resolve the problem.^30^ We found that 12 × 12-mm^2^ wide-field OCTA had better diagnostic ability for NPDR than a central 6 × 6-mm^2^ cropped field. Recently, Hirano and colleagues^31^ compared the discriminative power of DR presence in different scanning protocols (3 × 3-mm^2^, 6 × 6-mm^2^, and 12 × 12-mm^2^ fields), and their results suggest that 3 × 3-mm^2^ field images might best determine DR. The discrepancy with the present study could be partially explained by inclusion of patients with proliferative DR for which neovascularization in the periphery may confound the quantification of OCTA-based vascular metrics. Moreover, results may depend on the exact parameters of image acquisition including A-scan rate, B-scan rate, and oversampling density as well as the filters and algorithms used for quantification of vascular metrics.^32,33^

In the present study, we chose the approach of separating larger vessels from capillaries to assess the diagnostic performance of OCTA for DR. This approach was chosen because large vessels have a substantial contribution to the total peripapillary perfusion. Moreover, larger venular caliber and smaller arterial caliber are associated with DR progression and may therefore confound the association between capillary dropout and the stage of DR.^34,35^ Also, in the early stages of diabetes, retinal perfusion may be increased.^36,37^ In OCTA images, venules could be particularly important confounders for assessing DR because of their large diameters. Our results suggest that excluding the large vessels from perfusion density quantification provides extra sensitivity to NPDR diagnostic performance. A similar concept has been applied recently in calculating peripapillary perfused capillary density in patients with glaucoma^38^ and macula capillary density in patients with DR.^39^ Several studies^40,41,42^ have shown that subtraction of the peripapillary large vessels is associated with enhanced diagnostic accuracy of peripapillary OCTA for glaucoma. Moreover, Rosen et al^39^ detected early macular capillary changes in participants with diabetes without DR by removing the large vessels from the imaged area. By contrast, in our study, no improvement in diagnostic performance was found between the control group and the group with diabetes and no DR, most likely because of the good diabetes control and/or use of a wide-field approach in which macular vessel contributed little to the overall vascular metrics.

### Limitations

This study has limitations. Our results are based on a relatively small sample size. Participants with severe NPDR were combined with participants with moderate NPDR because of insufficient numbers. In this study, we excluded patients who had undergone laser photocoagulation. The practice pattern in many centers in Singapore is to treat patients with severe NPDR with photocoagulation early before the progression to proliferative DR. Therefore, the prevalence of untreated severe NPDR in our population was low. During clinical recruitment, 9 of 17 participants (53%) with mild NPDR (25 eyes [32.9%]) and moderate to severe NPDR (28 eyes [36.8%]) had already received laser treatment and were excluded from this analysis. In the national DR screening program, similar to many other developed country programs, the definition for referable DR is moderate or worse NPDR. Therefore, the stratification of moderate or worse NPDR from mild NPDR or no DR still represents an important clinical outcome.

The large vessel segmentation algorithm used in the present study was based on several vessel enhancement filters that assume a tubelike structure of the vessels. Segmentation errors with appearance of vessel branching, crossing, or vessel tortuosity were not evaluated systematically but appeared to be small. The ICC value was modest based on the small sample of older persons. The usability of the current algorithm needs to be further validated in a larger and more representative group of individuals. The initial quality check of wide-field OCTA excluded 30% to 40% of these scans because of multiple artifacts, such as low contrast, extensive motion artifacts, layer segmentation errors, and fixation problems. Exclusion of these scans might bring a bias to data selection because imaging may be more challenging for participants with worse visual acuity. The value of grading severity of NPDR is in the associated risk of progression to proliferative DR, which guides the management and follow-up of these patients.

This is a retrospective case-control study that provides no insight into how well the OCTA capillary defect correlates with DR progression. Progression of DR and diabetic macular edema are associated with features identified on fundus fluorescein angiography,^24^ but the association with OCTA metrics is not well established because there are limited longitudinal data from this recently developed technique. One study showed that OCTA metrics, especially in deep capillary plexus, predicted DR progression^43^ based on a small field of view (3 × 3-mm^2^ field). An OCTA with higher speed and better tracking systems as used in the present study may enable wide-field OCTA scan to be captured in a single shot and thus may show better predictive value for DR progression.

## Conclusions

Wide-field OCTA imaging may be useful for assessing peripheral capillary perfusion in eyes with DR. Isolation of large vessels from capillary perfusion density calculation may be associated with improved sensitivity to detect DR and may therefore be useful for disease prediction. The approach chosen in the present study may be particularly useful to detect peripheral capillary nonperfusion in patients with diabetes.
