# Grant Numbers Added to Funding/Support

**DOI:** 10.1001/jamanetworkopen.2020.10994

**Published:** 2020-06-05

In the Original Investigation, “Quantitative Microvascular Analysis With Wide-Field Optical Coherence Tomography Angiography in Eyes With Diabetic Retinopathy,”^1^ published on January 17, 2020, grants OFLCG/001/2017 and TA/MOH-0055/2017 from the National Medical Research Council were added to the Funding/Support section. This article has been corrected.
